# Therapeutic Communities for Addictions: A Review of Their Effectiveness from a Recovery-Oriented Perspective

**DOI:** 10.1155/2013/427817

**Published:** 2013-01-15

**Authors:** Wouter Vanderplasschen, Kathy Colpaert, Mieke Autrique, Richard Charles Rapp, Steve Pearce, Eric Broekaert, Stijn Vandevelde

**Affiliations:** ^1^Department of Orthopedagogics, Ghent University, H. Dunantlaan 2, 9000 Ghent, Belgium; ^2^Boonshoft School of Medicine, Center for Interventions, Treatment and Addictions Research (CITAR), Wright State University, 3640 Colonel Glenn Highway, Dayton, OH 45435, USA; ^3^Oxfordshire Complex Needs Service, Oxfordshire and Buckinghamshire Mental Health NHS Foundation Trust, Manzil Way, Oxford OX4 1XE, UK; ^4^Faculty of Education, Health and Social Work, University College Ghent, 9000 Ghent, Belgium

## Abstract

Therapeutic communities (TCs) for addictions are drug-free environments in which people with addictive problems live together in an organized and structured way to promote change toward recovery and reinsertion in society. Despite a long research tradition in TCs, the evidence base for the effectiveness of TCs is limited according to available reviews. Since most of these studies applied a selective focus, we made a comprehensive systematic review of all controlled studies that compared the effectiveness of TCs for addictions with that of a control condition. The focus of this paper is on recovery, including attention for various life domains and a longitudinal scope. We searched the following databases: ISI Web of Knowledge (WoS), PubMed, and DrugScope. Our search strategy revealed 997 hits. Eventually, 30 publications were selected for this paper, which were based on 16 original studies. Two out of three studies showed significantly better substance use and legal outcomes among TC participants, and five studies found superior employment and psychological functioning. Length of stay in treatment and participation in subsequent aftercare were consistent predictors of recovery status. We conclude that TCs can promote change regarding various outcome categories. Since recovering addicts often cycle between abstinence and relapse, a continuing care approach is advisable, including assessment of multiple and subjective outcome indicators.

## 1. Introduction

Drug addiction is a complex mental health problem that is often associated with difficulties in various life domains such as unemployment, homelessness, relational conflicts, problems with the courts, and psychiatric comorbidity [1, 2]. While some of these problems certainly evolve from the abuse of substances, many eventual addicts suffer from these problems prior to the onset of their drug use [3]. In both cases, drug addiction has generally been treated as an acute condition during brief episodes of residential care or several months of outpatient treatment, where the primary if not exclusive focus has been on abstinence to the exclusion of other concerns [4]. In contrast, addiction is increasingly regarded as a chronic relapsing disorder where recovery is possible [5], but often the one that requires intensive or even multiple treatment episodes and/or strong personal or community resources. A continuing care approach is needed to initiate and maintain recovery [6, 7]. The recovery movement focuses on individuals' perceived needs and objectives and sees abstinence as a potential resource, but not as a prerequisite, for recovery [8–10]. A recovery-oriented approach in addiction research implies attention for the evolutions on various life domains and individuals' subjective well being as well as the adoption of a longitudinal perspective to understand the complexity of individuals' substance use careers and recovery processes [11]. 

A wide range of treatment and support services are available for persons with alcohol or drug addiction problems: detox programs, drug-free outpatient treatment, methadone maintenance therapy, long-term residential treatment programs, and harm reduction services. Therapeutic communities (TCs) for addictions, also called drug-free or concept TCs, aim at the reinsertion into society of former drug addicts and were one of the first specialized treatment initiatives for individuals with addiction problems, that evolved outside—and often in reaction to—the traditional mental health care. The TC history dates back to Synanon, a self-supporting community of ex-addicts founded in 1958 in Santa Monica (California) [12]. A TC can be defined as “a drug-free environment in which people with addictive problems live together in an organized and structured way to promote change toward a drug-free life in the outside society” [13]. Until the mid-1980s, TCs had a predominant position in most Western addiction treatment systems, but due to the drug and HIV epidemic larger scale harm reduction initiatives (e.g., methadone maintenance, needle exchange programs) became the central focus of most West European drug policies. Despite the long-standing and worldwide availability of TC treatment, TCs were criticized for their limited coverage of drug addicts, the high costs of long-term residential treatment, and the lack of evidence of effectiveness resulting from randomized controlled trials. Moreover, high drop-out and relapse rates, altered client expectations and social norms and criticism on the impact of lengthy stays in closed communities further questioned the appropriateness of TC treatment around the turn of the century [14].

Although outpatient, medically-assisted (substitution) therapy is currently the most common addiction treatment modality [15, 16], one out of three clients in the European Union is engaging in other types of treatment, including therapeutic communities [17]. Recovery-oriented treatment in TCs starts from the widely accepted concept “community as a method” [18] and has been implemented on all continents. The standard TC model has been modified to address the needs of specific populations (e.g., women with children, persons with comorbid psychiatric disorders) or new phenomena (e.g., TCs in prisons, methadone substitution during TC treatment) in the so-called modified TCs (MTC) [19]. The TC method and objectives match well with the emerging recovery movement, since TC treatment can be regarded as an educational process where individuals are supported on their personal journey towards recovery and a drug-free lifestyle and to gain back control over their own lives [20]. 

Despite a long research tradition in TCs [21, 22], the evidence base for the effectiveness of TCs is limited according to the prevailing Cochrane hierarchy of scientific evidence [23]. Available reviews have been biased by a selective focus on some types of TCs or study designs and a predominant focus on drug abstinence. The frequently cited Cochrane review by Smith and colleagues [23] only included randomized trials, while random group allocation appeared to be either not feasible (i.e., significantly higher drop-out among controls) or advisable (i.e., motivation and self-selection are considered to be crucial ingredients of the treatment process) in several studies [24, 25]. Consequently, this meta-analysis included some studies without true randomization and excluded a large number of good quality quasi-experimental studies. A recent review by Malivert and colleagues [26] left out studies on prison TCs, while this type of modified TC has been the most frequently studied TC model during the last decade. Moreover, abstinence and treatment completion were the sole outcome measures in this study. Finally, the meta-analysis by Lees and colleagues [27] can be regarded as outdated, as it does not include any published study since 1999. 

Since sound scientific evidence is needed to inform service users, treatment providers, and policy makers about TCs' potential to promote recovery, the aim of this paper is to review the effectiveness of TCs for addictions, based on a comprehensive systematic review of available randomized and nonrandomized controlled studies. The paper is limited to studies with a controlled design, as these are robust study designs that generate a high level of evidence. Also, nonrandomized studies were included, since the number of randomized studies was very small (*n* = 5) and true randomization was compromised in several studies. Given the focus on recovery, a range of outcome measures—apart from abstinence—will be evaluated and a long-term outcome perspective will be applied, including an assessment of the influence of aftercare or continuing support. 

## 2. Methods

This narrative review focuses on controlled studies (randomized trials as well as quasi-experimental designs) of therapeutic communities for addictions. We opted for a narrative review instead of a meta-analysis, given the heterogeneity of the study methodologies and the variety in data reporting. Studies were eligible if they met the following inclusion criteria.

- Intervention: therapeutic communities for the treatment of drug addiction that are long-term hierarchically structured (residential) educational environments, where former drug users live together and work towards recovery, and which are based on self-help and mutual help principles [12, 21].
- Target population: adults addicted to illegal drugs (mostly heroin, cocaine, or amphetamines), often in combination with an addiction to other (legal) substances (e.g., alcohol, prescription drugs). Studies including persons with comorbid psychiatric disorders were eligible, if all study participants had a drug addiction.
- Outcome measures: at least one of the following (nonexhaustive) list of outcome measures was reported: substance use (illicit drug use, alcohol use), length of stay in treatment (retention, treatment completion/drop-out), employment status, criminal involvement, health and well being, family relations, quality of life, treatment status, mortality, and so forth. Objective (describing the actual situation) and subjective (indicating individuals' personal perspective) indicators were considered, as well as self-report measures, biological markers, and administrative data.
- Study design: randomized controlled trials and quasi-experimental studies that have compared prospectively residents that followed TC treatment with a control group that was treated in a usual care setting (“treatment as usual”/standard of care) or another type of TC (e.g., shorter program/day TC) or with a control group out of treatment (e.g., in prison/waitlist controls). Studies needed to report findings on TC outcomes separately from these of other types of interventions (e.g., aftercare).

Available reviews and meta-analyses were not included, but all studies selected for the reviews were screened based on the aforementioned inclusion criteria. Studies that did not focus on TC treatment, but on another type of residential care, were excluded from the paper. If several publications concerned the same baseline sample and study design, these publications were regarded as one single study.

### 2.1. Search Strategy

We searched the following databases: ISI Web of Knowledge (WoS), PubMed, and DrugScope, up to December 31, 2011. There were no language, country, or publication year restrictions. Search strategies were developed for each database, based on the search strategy developed for ISI Web of Knowledge, but were revised accordingly to take into account differences in controlled vocabulary and syntax rules. The key words we searched for were “therapeutic communit*” AND “drug* or addict* or dependen* or substance use” AND “outcome* or evaluation or follow-up or effectiveness.” The reference lists of retrieved studies and of available reviews were checked for relevant studies. In addition, the index of the International Journal of Therapeutic Communities, a specialized peer-reviewed journal on therapeutic communities and other supportive organisations, was screened for relevant publications.

**Figure 1:**
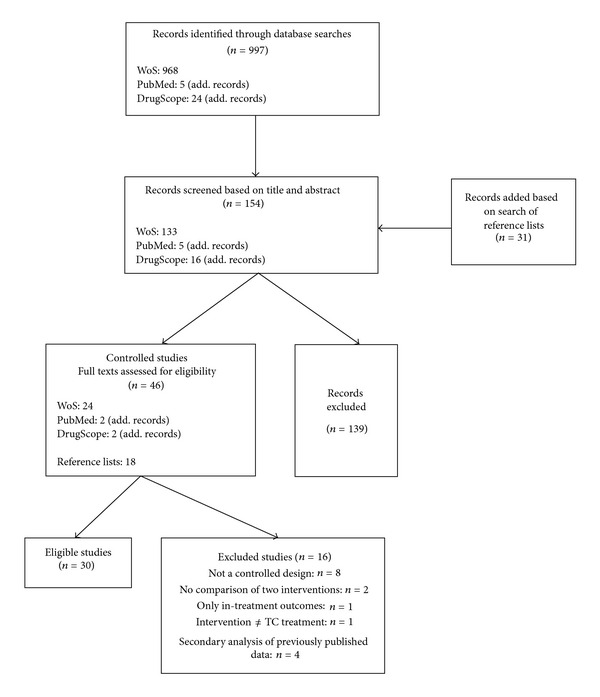
Flowchart of the search process and number of studies retained/excluded in each phase.

Our search strategy revealed 997 hits, which resulted in a first selection of 185 records, based on title and abstract (see Figure 1). Thorough analysis of these abstracts by two independent reviewers (Mieke Autrique and Wouter Vanderplasschen) led to the selection of 46 studies. 

In addition to the database search, conference abstracts of European Federation of Therapeutic Communities (EFTC), World Federation of Therapeutic Communities (WFTC), and European Working Group on Drugs Oriented Research (EWODOR) conferences and the grey literature were scanned for relevant (un)published studies. We made a search of the registry of ongoing clinical trials to identify any ongoing RCTs. In case a publication could not be tracked through the Ghent University online library system, the study authors were contacted for a copy of the original manuscript. Finally, TC experts in various countries as well as the European Monitoring Centre for Drugs and Drug Addiction (EMCDDA) national focal points were contacted to retrieve additional (un)published or ongoing studies that have assessed the effectiveness of TCs for addictions.

### 2.2. Study Selection

In total, 46 controlled studies were identified (28 based on the previously mentioned search strategy and 18 additional titles were selected based on the reference lists of selected studies and available reviews). After reading the full texts of these articles, 16 studies were excluded, because only in-treatment outcomes were reported (*n* = 1), because the treatment provided was not in line with the TC definition we put forward (*n* = 1), or because the study design was deemed not a controlled design (*n* = 8). Four studies were excluded as they concerned secondary analyses of previously published data, usually with a focus on a specific subsample. Two studies did not compare TC treatment with a control intervention but rather compared outcomes related to specific client characteristics. 

### 2.3. Data Extraction and Analysis

Two reviewers (Mieke Autrique and Wouter Vanderplasschen) extracted data on the characteristics and results from the selected studies into a large summary table (cf.  Table 1). The following study characteristics were extracted: (1) author, country (state), and year of publication; (2) type of study design and timing of follow-up measurements; (3) inclusion criteria and characteristics of the study participants + attrition rates at follow-up; (4) type of TC (including length of treatment) and type of control condition; and (5) outcome categories: retention and completion rates, substance use outcomes (drug and alcohol use), criminal involvement, employment, and other outcomes like health status, housing situation, and a column including determinants/correlates of abstinence/retention. Findings from studies including multiple follow-up assessments were grouped and numbered accordingly (cf.  Table 1). We compared reported outcomes in various categories at all reported follow-up moments post treatment (cf.  Table 2.). In this summary table, “+” indicates a significant difference regarding the outcome category in favor of the experimental condition, while “-” indicates a significant difference in favor of the control group. “=” means that no significant between group differences were reported; alternatively text can be rephrased as follows: that no significant differences were reported between the experimental and the control group.

## 3. Results

Based on our review of controlled studies of TC effectiveness, we identified 30 publications that included a longitudinal evaluation of TCs for addictions and applied a prospective controlled study design (cf.  Table 1). These 30 publications are based on—in total—16 original studies, since several articles referred to the same (large) study and/or to various measurements regarding one single study (e.g., the Delaware study (no. 7) by Inciardi and colleagues [28–32]; the Amity prison study (no. 8) by Prendergast and colleagues [33–35]). Thorough methodological screening revealed that only five studies could be regarded as truly randomized (cf.  Table 1), since in most studies the random group allocation process was compromised at some point [25, 36] or was not possible/advisable at all [24, 37, 38]. The methodological quality of the studies varied but was often rather poor due to high attrition rates, lack of objective verification of study findings, and a focus on one single study site (cf.  Table 1). The oldest controlled studies date back to the beginning of the 1980s [39–41]. The bulk of studies has been carried out/published in the 1990s. All controlled studies have been performed in the United States. Despite a growing research tradition in Europe, Australia, and South America, only observational uncontrolled studies have been carried out on these continents.

The follow-up period in most controlled studies is between 6 and 24 months, and only three studies have followed participants for more than 36 months. Study outcomes may vary according to the follow-up moment [24, 25, 33], but usually the magnitude of the difference(s) between the experimental and control group diminished over time (cf.  Table 2). Overall, great within-group reductions in problem severity were observed between baseline and follow-up assessments, in particular regarding drug use, criminal involvement, and employment. The two outcome measures that were assessed in most studies are “substance use” and “criminal involvement.” All included studies reported at least one outcome measure in one of both categories. Eight out of 13 (note that this number is lower than 16, as not all studies reported outcomes concerning all categories) studies reported at least one positive significant difference between the TC and control group regarding legal outcomes at the one-year follow-up, while 9/14 studies found significantly better substance use outcomes among the TC group at that time (cf.  Table 2). All studies included multiple outcome indicators (also within one category), but only one study succeeded to find several significant, positive outcomes regarding most legal outcome measures (i.e., reincarceration rate, days to first illegal activity/incarceration, and length of prison sentence) [34]. Most studies found only one significant between group difference per category (e.g., time to drug relapse), while other outcome indicators within this category did not differ between groups (cf.  Table 1). Significantly better outcomes in one category (e.g., substance use, criminal involvement) are not necessarily accompanied by improved outcomes on other domains (e.g., employment, psychological health). Only four studies found significant differences regarding three or more outcome categories [24, 32, 42, 43].

### 3.1. Treatment Retention, Health, and Social Functioning

As opposed to all other outcome categories, TC participants scored worse in comparison with controls on treatment retention/completion. Only two studies showed higher retention rates for the TC group, while three studies found significantly worse completion rates among TC-participants, and six studies found non-significant between group differences, mostly in favor of the control condition (cf.  Table 1). Substantial drop-out has been observed in most long-term TC programs, especially in the early phases of treatment [48]. Studies that have compared longer and shorter TC programs usually found lower completion rates in longer and more intensive programs [38, 51]. 

Five out of six studies that have reported employment outcomes found significantly better employment rates among TC participants. Also, five studies (out of 7) showed superior outcomes on psychological symptoms, as compared with controls. Other outcomes that were studied are risk behavior (*n* = 1) and family and social relations (*n* = 2), which were found to be better in two studies [32, 48].

### 3.2. Substance Use Outcomes

Although TC participants had at some point posttreatment better substance use outcomes than controls in 10 studies, substance use levels varied greatly and overall, between 25% and 55% of the respondents relapsed to drug use after 12 to 18 months. Some studies found very low initial relapse rates (e.g., 4% [38], 9% [42] and 15% [43]), while others found much higher relapse rates (e.g., 53% [34] and 69% [29]). Usually, time to relapse was longer among TC participants [52]. One of the few controlled studies that followed prison TC-participants up to three years after their release found a relapse rate of 77% in the TC and 94% in the control condition [29]. Lower relapse rates were usually associated with longer treatment exposure (length of stay in treatment/retention) [24, 31, 39, 41, 52] and participation in subsequent treatment or aftercare [32, 35]. Treatment drop-out and relapse after treatment were predicted in at least two studies by the severity of substance use at baseline [28, 38].

### 3.3. Legal Outcomes

The majority of studies found a positive impact of TC treatment on diverse legal outcomes, such as recidivism, rearrest, and reincarceration. Recidivism rates (self-reported criminal involvement) of TC participants after one year are usually around 40%–50% [19, 31], as well as rearrest rates [29, 44], although one study reported a rearrest rate of only 17% 18 months after the start of TC treatment [42]. Reincarceration rates 12 to 18 months after release are between 30% and 55% in most studies, although Sacks and colleagues have reported clearly lower rates (19% and 9%, resp.) in two studies [19, 36]. Long-term follow-up measurements of prison TC participants indicate rearrest rates of 63% after three years [29] and 80% after five years [44] and reincarceration rates of over 70% after 5 years [33, 44]. Again, time to reincarceration was lower in the TC group and treatment completion and/or time in treatment predicted absence of recidivism [28, 31, 33, 36, 42, 49]. Treatment completion was found to be associated with (older) age, single (instead of poly) drug dependence and being on parole [42].

### 3.4. Long-Term Outcomes and Outcome Predictors

Six controlled studies have investigated the outcomes of TC participants in comparison with controls beyond a period of 12 to 18 months (cf.  Table 2). Five of these studies show significantly better legal outcomes in favor of the TC group, while only three studies could demonstrate significantly lower levels of illegal drug use two years after TC treatment. One of these studies [40] found a higher prevalence of alcohol problems among TC participants at the two-year follow-up, when compared with controls who only followed a short detoxification period. 

Several studies have identified correlates of relapse and recidivism after TC treatment. Participation in aftercare [28, 35, 44], posttreatment employment [37], and older age [28, 33] were found to be the most common predictors of abstinence and absence of rearrest (cf.  Table 1). The effectiveness of completing treatment was shown in several studies, as TC + aftercare completers had better outcomes than aftercare drop-outs, who had in turn better outcomes than TC completers and TC drop-outs [33, 35]. Martin and colleagues [32] even found no differences between inmates who followed in-prison TC treatment without subsequent aftercare and controls who received usual work release. Relapse to drug use is often associated with reoffending and reincarceration [46]. 

### 3.5. Type of Controls and TC Modalities

Eleven studies have compared TC treatment with some form of usual care (e.g., case management, standard treatment, and probation), and five studies compared one type of TC with another form of TC treatment (modified versus standard TCs, or short versus long TC programs). In the latter case, the longest/most comprehensive TC program was regarded as the experimental condition, while the shorter/least intensive program was seen as the control condition. Only three comparisons of longer and shorter TC programs yielded significantly better substance use outcomes at the first follow-up moment [25, 41, 42], while overall few significant differences were observed in comparison with other TC modalities. Two studies found better employment outcomes compared with lower intensity TC models, and one study found fewer psychological symptoms and relational problems among the higher intensity treatment group. Some studies have included multiple control conditions [29], but usually significant differences were only observed when the most intensive intervention was compared with the least intensive treatment condition.

Most controlled studies of TC effectiveness have focused on TCs in prison settings (*n* = 9) that prepare inmates for reintegration in society, while seven studies concerned TCs in the community. Whereas a substantial number of residents enter community TCs under legal pressure, TC treatment in prison can be regarded as a different context given the compulsory custody and conditional release term and privileges. Substance use outcomes in community TCs were significantly better than those among controls in five (out of 6) studies, while legal outcomes were found to be superior in three (out of 4) studies of community TCs. On the other hand, only in four (out of 7) studies of prison TCs, the experimental group scored significantly better than the control group, and only one study could demonstrate this difference beyond the one-year follow-up assessment [28]. Six (out of 9) prison TC studies found significantly better legal outcomes among TC participants. Three studies could demonstrate these gains after two years, and two studies found these benefits maintained up to five years after prison TC treatment [28, 33].

## 4. Discussion

### 4.1. Main Findings

This narrative review was based on 16 studies that have evaluated the effectiveness of TCs as compared with other viable interventions regarding various indicators related with recovery: substance use, criminal involvement, employment, psychological well being, and family and social relations. Based on the study findings, we can conclude that there is some evidence for the effectiveness of therapeutic community treatment. Almost two out of three studies have shown significantly better substance use and legal outcomes at the first follow-up moment after treatment among persons who stayed in a TC as compared with controls. Five studies found superior employment outcomes among TC participants, while another five studies showed significantly fewer psychological problems in the experimental group. Only four studies have reported significantly better differential outcomes in at least three outcome categories. This does not mean that TC participants do not improve equally on all life domains, but these outcomes often remained unreported or the observed progress did not differ significantly from that among the control group. Several reviews [22, 26, 27, 54] have addressed the question whether TCs generate better outcomes than other interventions, often leading to conflicting and not really convincing conclusions. Although several studies included in this paper showed improved differential outcomes [28, 33, 38, 39, 42, 50], these findings were observed among varying populations in diverse settings, and few studies have succeeded to replicate the findings from other studies in exactly the same conditions. Moreover, some studies [25, 42, 45] have compared modified TCs with standard TCs that were not specifically adapted to address the needs of special target groups. In general, such comparisons of one type of (modified) TC with a less intensive (standard) TC model did not demonstrate much between group differences, given the strong similarities between both treatment conditions. Consequently, the main question is not whether one type of TC is better than another intervention/type of TC, but rather which persons benefit most from (what type of) TC treatment at what point in the recovery process [22]. Also, uncontrolled treatment outcome studies have repeatedly shown fairly similar effects of various types of residential treatment [55, 56] and when compared with outpatient methadone treatment [40, 57, 58], demonstrating that—from a longitudinal perspective—no single intervention is superior to another. Not the differential effectiveness of TCs, but rather individuals' assets and community resources and their personal needs and goals will determine whether TC treatment is indicated on the road to recovery. 

### 4.2. Towards a Recovery Perspective on TC Treatment

While looking beyond abstinence and desistance is warranted from a recovery perspective [8], six of the selected controlled studies did not report other than substance use and legal outcomes. Stable recovery in opiate addicts has been primarily associated with social participation and having meaningful activities and purposes in life, rather than with drug abstinence or controlled drug use [59]. Focus groups with drug users regarding their perceived quality of life revealed few specific but mostly generic aspects of QoL like well being, social inclusion, and human rights [60]. Still, a predominant focus on objective socially desirable outcome measures (e.g., work, alcohol and drug use, and recidivism) prevails in addiction research, while more subjective outcome indicators like emotional well being, quality of life, or job satisfaction have largely been disregarded [61]. Such a broad perspective is also needed in TC research, as it allows a more accurate evaluation of individuals' personal growth and well being after TC treatment. Up to now, recovery has primarily been measured based on abstinence rates after TC treatment, while abstinence is not a synonym of nor a prerequisite for recovery [8]. Total abstinence—as required during and expected after TC treatment—appears not to be self-evident, not even after a lengthy treatment episode in a TC and subsequent continuing care. TC participants typically improve on most life domains during the first months of treatment and are usually able to maintain this status until they leave treatment [26, 48]. However, once individuals leave the TC, success rates tend to drop quickly, especially during the first month(s) after treatment. A recent review of longitudinal (mostly uncontrolled) TC studies showed that 21% to 100% relapsed into drug use six months to six years after leaving treatment [26]. We found substantial relapse rates (25%–70%) 12 to 18 months after leaving treatment, which indicate that 30% to 75% of the studied TC sample did not relapse within one year after TC treatment. Although the definition of “relapse” varied largely between studies (e.g., any substance use, illicit drug use, regular use, and last month use), relapse can be addressed in at least two different ways, depending whether one starts from an acute or a continuing care perspective. The former approach sees relapse as a failure as treated individuals did not succeed to abstain from drug use after intensive treatment. The latter perspective acknowledges the chronic relapsing nature of drug addiction and assumes that relapse is part and parcel of the recovery process and should rather be considered as a learning moment to keep the precarious balance between abstinence and relapse [62]. Factors that may contribute to recovery are longer length of stay in the TC (retention) and participation in subsequent aftercare, since both variables have been consistently identified as predictors of improved substance use outcomes [23, 26]. Surprisingly, treatment completion was not found to be a predictor of abstinence, but it was associated with reduced recidivism rates in several studies of prison TCs [33, 36]. 

Treatment in TCs for addictions takes time, usually around 6 to 12 months, which heightens the possibility that residents leave prematurely [27]. Retention in (longer term) TCs is typically lower than in shorter term programs [42, 51, 55], but in general TC residents who stayed longer in treatment had significantly better outcomes than persons who dropped out early. This has led to concerns with enhancing retention through the involvement of the family and social network and the use of senior staff [63] and with promoting initial engagement through motivational interviewing, contingency management, and induction interventions [64–66]. An alternative promising way of looking at retention may be to see it as the sum of treatment episodes in different services and the accumulation of associated treatment experiences instead of defining retention as a single uninterrupted stay in one treatment program [67]. Reentry in the community appears to be a critical point after TC treatment, if not prepared adequately (e.g., by providing aftercare) or if drug users go back to their old neighborhoods [68]. Some type of continuing support is warranted after TC treatment not only to prevent relapse, but also to link with employment/training and to engage in community-based activities. Moreover, treatment discharge should be dealt with in a flexible and individualized way, since some persons will need to be further supported or to reenter the community if they are doing poorly. 

The recovery movement starts from a longitudinal approach to addiction and other mental health problems [69], but few controlled studies have assessed TC outcomes beyond a two-year follow-up period. Available studies suggest that—despite a fading effect of TC treatment over time—recidivism rates continued to be significantly better than these of controls in three studies of prison TCs [28, 33, 37], while findings regarding substance use outcomes indicated fewer between group differences. The three-year follow-up outcomes of the Delaware prison study showed a 94% relapse rate among the usual care group (traditional work release) compared with a 77% relapse rate in the prison aftercare TC group [29]. These figures do not only illustrate the relapsing nature of addiction problems, but also point at the relatively poor effectiveness of treatment programs. Although robust study designs including substantial follow-up periods that are able to retain most respondents in the analyses are needed, one may not overestimate the lasting effects of one single (prolonged) treatment episode. Recovery is considered to be a lengthy process, and continuing care is needed to maintain recovery that has been initiated during, for example, TC treatment. Some studies have shown that the provision of aftercare was as or even more effective than initial TC treatment [29, 70], and the combination of TC treatment and subsequent aftercare has generated the best results [33, 71]. 

Finally, the study findings show that TC treatment has generated beneficial outcomes in diverse treatment settings and may have particularly strong effects among severely addicted individuals like incarcerated, homeless, and mentally ill drug addicts [22, 36, 37, 46]. Therefore, treatment in TCs should be considered as a specific intervention, reserved for drug addicts with multiple and severe problems. Although outpatient methadone maintenance therapy is the mainstream addiction treatment worldwide, therapeutic communities for addictions can be regarded as a valuable alternative for persons who do not do well in outpatient treatment due to the lack of structure and supports in the community and the fact that they live in neighborhoods that are pervasively affected by drug abuse [68]. TCs can be supportive places where clients can learn some of the internal control and refusal skills conducive to stable recovery. Motivation, social support and coping with stress without using substances appear to be key factors in successful recovery [72]. 

### 4.3. Limitations of the Paper

First, most selected studies were published in peer reviewed journals. Although the restriction of peer-review guarantees some form of quality control, it may have induced a selection bias as the likelihood of retrieving non-English language articles was limited in this way. Only results that were reported in the published papers could be included, while it was often unclear whether the nonreporting of some specific outcomes (e.g., recidivism, alcohol use) meant that this information was not collected, not analyzed, or did not yield significant findings. Second, substantial heterogeneity has been observed between the included studies, not only regarding program and setting characteristics, but also regarding sample characteristics and outcome measures. Despite the common “community as method” principle [18], TCs for addictions consist of various practices and programs with varying treatment length. Standard and modified TC programs have been evaluated in this paper, as well as TCs in prison settings and aftercare TCs. This heterogeneity should be taken into account when interpreting the study outcomes. Although the underlying elements may be fairly similar across TC programs, the dosage of the program and fidelity to the concept may have varied considerably [22, 27]. Also, types of controls varied across studies from waitlist controls to interventions that differed only slightly from the experimental group (e.g., residential/longer versus day/shorter TC programs). Another limitation is the use of varying outcome measures and instruments across studies, which further hampers the replication and generalization of the findings. Third, this systematic review was not restricted to randomized trials, although the Cochrane collaboration and other proponents of the evidence-based paradigm regard this type of study design as the gold standard for the evaluation of evidence of effectiveness [73]. Given the difficulties to apply this design to long-term and comprehensive multi-interventions like TCs and the low number of randomized controlled trials on the effectiveness of TCs, a comprehensive review of randomized and nonrandomized controlled studies was deemed to be of surplus value in comparison with available reviews, still generating an acceptable level of evidence [68]. Treatment drop-outs may further compromise the validity of the reported results. Several studies only included substance users who stayed for a substantial period in the TC or who completed treatment but made no intent-to-treat analysis of everyone who started TC treatment (cf.  Table 1). Finally, this is a narrative review of controlled studies that does not allow to weigh the findings from different studies or to estimate effect sizes. A meta-analysis was not possible at this point, given the substantial heterogeneity between programs and the diverse outcome categories and measures that were reported in the selected studies.

## 5. Conclusion

Therapeutic communities for addictions can be regarded as recovery-oriented programs that produce change regarding substance use, legal, employment, and psychological well-being outcomes among drug addicts with severe and multiple problems. Despite various methodological constraints, TCs appeared to generate significantly better outcomes in comparison with other viable interventions in two out of three studies. TC programs have usually been evaluated from an acute care perspective with a primary focus on abstinence and recidivism, while a continuing care approach including multiple and more subjective outcome indicators is necessary from a recovery perspective. If residents stay long enough in treatment and participate in subsequent aftercare, TCs can play an important role on the way to recovery. Abstinence may be just one resource to promote employment or enhance personal well being which can in turn contribute to recovering addicts' participation in community-based activities and their social inclusion.

## Figures and Tables

**Table 1:** Overview of included studies (*n* = 30).

Authors	Study design + measurement(s)	Participants	Intervention + comparison group	Outcome measures					Correlates of relapse/abstinence
				Retention	Substance use	Crim activity	Employment	Other
*Author names,* *year of publication,* *and place (country + state)*	*Type of study (RCT, quasi-experimental, etc.)* *Length of followup period, for example, outcomes 12 months after discharge*	*Number and type of participants* *Attrition rate: how many of the original participants retrieved at followup?* *Specific inclusion criteria*	*Type of TC (traditional, modified, prison TC) + type of control condition* *Treatment length*	*For example, completion rates,* *months in Tx*	*For example, abstinence/ * *relapse rates * % *use in last month * *time to first use after Tx *	*For example, * *reconviction/rearrest rates * *ASI scores * % *reincarceration *	*For example, * % *employment *	*For example, * % *homeless * % *psychiatric disorders * *quality of life * *family relations *	*Which variables were correlated with improved outcomes?*

(1) Sacks et al., 2012 (Colorado, USA) [19]	Prospective controlled study design (partial randomisation, since assignment ratio changed during the study) Outcomes 12 months after TC-entry	127 male offenders with substance use and mental disorders who participated in various types of prison Tx FU: 86.6%	Reentry MTC (*n* = 71) 6 month program Controls: parole supervision case management (*n* = 56)			SR drug offences: 37 versus 58%*; reincarc. rate: 19 versus 38%* SR crim activity: 39 versus 62%*; days till reincarc.: 161 versus 168*			

(2) Zhang et al., 2011 (California, USA) [44]	Prospective controlled study design (QES) Outcomes 1 and 5 years after prison release	798 male offenders with documented history of substance abuse FU: 100% (data from official records)	Prison-based TC (*n* = 395) 18 month program Controls: matched group of untreated inmates in nearby prison (*n* = 403)		1 year FU	Rearrests: 54.0 versus 47.6% (ns); reincarc.: 54.7 versus 51.9% (ns); days in prison: 79.1 versus 77.4			Participation in aftercare mediated reincarc. rates (after 1 year (ns), not after 5 years) and time in prison (after 1* and 5 years (ns))
					5 year FU	Rearrests: 80.4 versus 78.2% (ns); reincarc: 72.4 versus 72.5% (ns); days in prison: 450.4 versus 412.7			

(3) Messina et al., 2010 (California, USA) [45]	Prospective randomised controlled study design Outcomes 6 and 12 months after release	115 female offenders with documented history of substance abuse FU: 83% after 6 months FU: 76% after 12 months	Gender-responsive MTC in prison (*n* = 60) 6 month program Controls: standard prison TC (*n* = 55) 6 month program	Months in aftercare: 2.6 versus 1.8* MTC group had higher OR (4.60*) of successful aftercare completion	No *≠* regard. alcohol and drug ASI composite scores + self-efficacy	No *≠* in (time to) return to custody: 31 versus 45%		No *≠* regard. family and psychological ASI composite scores	Greater reduction in drug use among MTC group*, when controlling for race, employ. + marital status Return to custody less likely among MTC group, when controlling for race, employ. + living status

(4) Welsh, 2007 (Pennsylvania, USA) [37]	Prospective controlled study design (QES in 5 state prisons) Outcomes up to 2 years after release (on average after 17 months)	708 male inmates admitted to drug Tx in prison FU: 100% (based on official records)	5 prison TCs (*n* = 217) Length varied from 9 (*n* = 1) to 12 (*n* = 3) and 16 months (*n* = 1); controls (*n* = 491): 3 other types of drug Tx (drug education, outpatient Tx, self-help groups)		No *≠* in number of pos. drug tests (35 versus 38%)	Lower reincarc and rearrest rates, respectively 30 and 24% versus 41 and 34%* Reincarc. and rearrest respectively 1.6* and 1.5* times higher among controls	Higher employ.: 39.2 versus 25.9%***		Reincarc. predicted by post-release employment status Drug relapse predicted by age and employment (older and employed persons less likely to relapse)

(5) Sullivan et al., 2007 (Colorado, USA) [46]	Prospective randomized controlled study design Outcomes 12 months post-release	139 male inmates with substance use and other psychiatric disorders FU: 75% (82 versus 69%)	Prison MTC (*n* = 75) 12 month program Controls: standard mental health Tx in prison (*n* = 64)		Rates of any substance use: 31 versus 56%**; any illicit drug use: 25 versus 44%*; alcohol intox.: 21 versus 39%* Time to relapse: 3.7 versus 2.6 months*				Sign. association between relapse and committing new (nondrug) offences Increased OR for reoffending (4.2*) and reincarc. (5.8*) among persons who relapsed in substance use

(5) Sacks et al., 2004 (Colorado, USA) [36]	Prospective controlled study design (no true randomisation, since 51 subjects moved from one condition to another) Outcomes 12 months after prison release	185 male inmates with substance use and other psychiatric disorders FU: 75% (82 versus 69%)	Prison MTC (*n* = 92) 12 months Controls: standard mental health Tx (*n* = 93)			Lower reincarc. rates: 9 versus 33%**; no *≠* regarding other criminal outcomes			MTC aftercare participants had superior outcomes regarding rates of reincarc.*, crim. activity* and drug-related crim activity* compared with controls Time in Tx predicted absence of reincarc** and crim activity**

(6) Morral et al., 2004 (Los Angeles, USA) [47]	Prospective controlled study design (cases assigned by probation) Outcomes 12 months after start TC program	449 adolescent probationers with substance abuse problems FU: 90.4% after 3 months FU: 91.3% after 6 months FU: 90.8% after 12 months	MTC in prison (Phoenix Academy) (*n* = 175) 9 month program Controls (*n* = 274): alternative probation disposition (res. group homes)	No *≠* in program retention	Improved substance use outcomes on substance problem index*, density index*, and involvement scale*	Greater, nonsign. declines on various measures of crim involvement		Greater reduction of somatic** and anxiety* symptoms + sign. larger reductions in psychological symptoms between 3 and 12 month FU	

(7) Inciardi et al., 2004 (Delaware, USA) [28]	Prospective controlled study design (group assignment by correctional staff) Outcomes 42 and 60 months after baseline	690 male inmates with substance abuse problems, eligible for work release FU: 69.8% after 48 months FU: 63.8% after 60 months	Work-release (transitional) TC (*n* = 472) 6 month program Controls: standard work release, without Tx (*n* = 218)		TC participation strongest predictor of drug-free status after 42 (OR 4.49***) and 60 months (OR 3.54***)	TC participation strongest predictor of absence of rearrest after 42 (OR 1.71**) and 60 months (OR 1.61*)			Older age predicted drug-free** and no rearrest status***, while frequency of prior drug use predicted relapse** after 48 months No previous Tx experience predicted relapse after 60 months, while older age* and being female* predicted no rearrest TC completion associated with no rearrest and being drug free after 42 and 60 months, with superior outcomes for persons who attended additional aftercare

(7) Martin et al., 1999 (Delaware, USA) [29]	Prospective controlled study design (partial randomization, since KEY program autom. followed by CREST) Outcomes 6, 18, and 42 months after baseline (i.e., 1 and 3 years after TC period)	428 inmates with drug abuse problems FU: approx. 80%	Transitional TC (CREST) (*n* = 157) 6 month program Controls: prison TC (KEY) (*n* = 38) 12 month program Prison TC + transitional TC (*n* = 68) Regular work release (*n* = 165)	18 month outcomes	31% drug-free versus 16%* in work-release group (versus 47% in KEY + CREST group)	57% not rearrested versus 46%* in work-release group (versus 77% in KEY + CREST group)			While greater exposure to TC Tx led to better outcomes after 1 year, at 3 years after discharge no *≠* were found between various TC modalities CREST drop-outs as likely to be rearrested as work-release group, but CREST completers** + CREST-completers who followed subsequent aftercare*** were least likely to be arrested CREST drop-outs more likely to be drug free than work-release group*, but CREST completers** + CREST-completers who followed subsequent aftercare*** even more likely to be drug free
				42 month outcomes	OR for being drug free 8.2 times higher in CREST-group**, 7.4 times in KEY-group**, and 6.7 times in KEY-CREST group* compared with work release group; 23% drug-free versus 6%* in work-release group	37% not rearrested versus 30% in work-release group after 42 months			

(7) Lockwood et al., 1997 (Delaware, USA) [30]	Prospective controlled study design Outcomes 6 months post-release	483 inmates with history of substance abuse FU: approx. 80%	Transitional TC (CREST) (*n* = 193) 6 month program Controls: prison TC (KEY) (*n* = 44) 12 month program Prison TC + transitional TC (*n* = 34) Regular work release (*n* = 212)		87% drug-free, versus 71% in KEY, 73.7% in work release and 93.3% in KEY-CREST group	86.5% no arrest, versus 75% in KEY, 59.9% in work release and 97.1% in KEY-CREST group			

(7) Nielsen et al., 1996 (Delaware, USA) [31]	Prospective controlled study design (QES) Outcomes after 6 and 18 months	689 inmates with history of substance abuse FU: 77 versus 72.6% after 6 months FU: 58.5 versus 36.7% after 18 months	Transitional TC (CREST) (*n* = 248) 6 month program Controls: conventional work release (*n* = 441)		Sign. lower relapse after 6 (16.2 versus 62.2)*** and 18 months (51.7 versus 79%)***	Sign. lower recidivism after 6 (14.7 versus 35.4)*** and 18 months (38.2 versus 63%)***			Age, race, and gender do not affect outcomes, but length of time in program reduced relapse and recidivism rates (ns) Program completion associated with fewer relapse*** after 6 and fewer recidivism after 6*** and 18 months*

(7) Martin et al., 1995 (Delaware, USA) [32]	Prospective controlled study design (QES) Outcomes 6 months after release	483 inmates with history of substance abuse FU: approx. 80%	Transitional TC (CREST) (*n* = 176) 6 month program Controls: prison TC graduates (KEY) (*n* = 43) Prison TC + transitional TC (*n* = 32) Regular work release (*n* = 206)		Probability of being drug free the highest among CREST (0.84)*** and KEY + CREST group (0.94)***	Prob. of being arrest free the highest among CREST (0.86)*** and KEY + CREST group (0.97)***		Prob. of no longer injecting the highest in CREST (0.97)*** and KEY + CREST group (0.97)	No *≠* between TC only and work release group on any of the outcome measures Longer time in (subsequent) Tx the best predictor of drug-free*** and arrest-free* status after Tx, as well as participation in a longer TC program

(8) Prendergast et al., 2004 (California, USA) [33]	Prospective randomized controlled study design Outcomes 5 years after release	715 male inmates with substance abuse problems FU: 81.2%	Amity prison TC (*n* = 425) 9–12 month program Controls: no Tx condition (waitlist) (*n* = 290)	Months receiving Tx post-release: 4.6 versus 1.7***	Heavy drug use past year: 24.9 versus 22.6%	Reincarcerated within 5 years: 75.7 versus 83.4%* Days to reincarc: 809 versus 634***	Stable job in past year: 54.8 versus 52.3%	Psychologic. distress: 31.8 versus 44.6	Reincarc. predicted by younger age* and fewer months in Tx after release*** Completion of TC and aftercare predicted absence of reincarc.***

(8) Prendergast et al., 2003 (California, USA) [34]	Prospective randomized controlled study Outcomes 12 months after release	715 male inmates with substance abuse problems FU: 74%	Amity prison TC (*n* = 425) 9–12 month program Controls: no Tx condition (waitlist) (*n* = 290)		Longer time to first drug use: 77 versus 31 days*** No *≠* in pos. drug tests (52.9 versus 61%)	1 year reincarc. rate: 33.9 versus 49.7%*; more days to first illegal act. (138 versus 71 days)***; no *≠* in type of arrest; more days to first incarcer. (285 versus 243 days)***; fewer months in prison (3 versus 4.7)***			Participation in Tx associated with more days to reincarc. Aftercare completers had the lowest reincarc rate + the longest time to first illegal activity and to reincarceration and fewer days in prison Prison TC drop-outs had the shortest time to SR drug use (32 days), followed by TC completers (62 days), aftercare dropouts (91 days), and aftercare completers (184 days) + more pos. drug tests

(8) Wexler et al., 1999 (California, USA) [35]	Prospective randomised controlled study design Outcomes 12 and 24 months after release	715 male inmates who volunteered for TC treatment in prison FU: 100% after 12 months FU: 36.8% after 24 months	Amity prison TC (*n* = 425) 8–12 month program Controls: no Tx condition (waitlist) (*n* = 290)			Lower reincarc rates after 12 (33.9 versus 49.7%)*** and 24 months (43.3 versus 67.1%)*** More days to reincarc after 12 (192 versus 172*) months OR for reincarc sign lower: 0.52*** after 12 and 0.63** after 24 months			Reincarc. rates sign lower after 12 and 24 months among TC + aftercare completers, as opposed to persons who dropped out previously Aftercare completion positively related to time to reincarc. + the strongest predictor of positive outcomes

(9) Greenwood et al., 2001 (San Francisco, USA) [25]	Prospective controlled study design (only partial randomisation, since sign. drop-out among control before Tx start) Outcomes 6, 12, and 18 months after admission	261 substance abusers seeking treatment at Walden House FU: 82.4% at 6 months FU: 82.7% at 12 months FU: 82.7% at 18 months	Residential TC (*n* = 147) 12 month program Controls: day TC program (same TC, but returned home at the end of the day) (*n* = 114)	Time in program: 109.8 versus 102.7 days	Total abstinence after 6 (62.6 versus 47%), 12 (47.9 versus 49%) and 18 months (50.4 versus 55.2%) OR for relapse at 6 months = 3.06*, not sign. at 12 and 18 months				Relapse after 18 months predicted by employment status prior to Tx start***, injecting drug use** and having >1 sexual partner**

(9) Guydish et al. 1999 (San Francisco, USA) [48]	Prospective controlled study design (only partial randomisation, since sign. drop-out among controls before Tx start) Outcomes at 6, 12, and 18 months	188 substance abusers seeking treatment at Walden House who participated in all 3 FU-interviews	Residential TC (*n* = 99) 12 month program Controls: day TC program (same TC, but returned home at the end the day) (*n* = 89)	No *≠* in time to drop-out (119.7 versus 108.1 days) 12 month retention in day TC: 17% versus 9%				Lower SCL scores at 6**, 12*, and 18* months, lower BDI scores after 12 months*, higher social support scores at 18 months*; lower social problem severity (ASI)*	Most changes observed during first months of Tx, followed by maintenance of change

(9) Guydish et al. 1998 (San Francisco, USA) [49]	Prospective controlled study design (only partial randomisation) Outcomes at 6 months	261 substance abusers starting treatment at Walden House FU: 82.4% at 6 months	Residential TC (*n* = 114) 12 month program Controls: day TC 12 month program (*n* = 147)	Tx adherence after 6 months: 29 versus 34% in day TC; Time in Tx: 109.8 versus 102.7 days				Lower ASI severity scores for social* and psychological problems**	Persons who stayed >6 months in Tx had sign lower legal, alcohol, drug, and social severity scores Alcohol severity reduced sign. if persons stayed >6 months in residential TC

(10) Nemes et al. 1999 (Washington, USA) [42]	Prospective, randomised controlled study design Outcomes 18 months after admission	412 substance users seeking Tx at a central intake unit FU: 93%	Standard TC (*n* = 194) 12 month program (10 months inpatient, 2 outpatient) Controls: abbreviated TC (*n* = 218): 12 month program (6 month inpatient, 6 month outpatient + extra services)	Completion rates: 33 versus 38% (ns), and similar time in Tx (8.2 versus 8.6 months)	Lower SR heroin use: 9 versus 15%*	Lower rearrest rates: 17 versus 26%** + longer time to arrest (9.4 versus 6.9 months)*	Employment rate higher in standard TC: 72 versus 56%**		Lower heroin and cocaine use levels + lower rearrest rates among treatment completers versus noncompleters Positive cocaine tests were associated with premature Tx drop-out Treatment completion was predicted by age, single heroin dependence, and parole status

(11) De Leon et al., 2000 (New York, USA) [24]	Prospective controlled study design (QES: sequential group assignment) Outcomes 12 and on average 24 months after baseline	342 homeless mentally ill substance abusers FU: 68% at 12 months FU: 82% at latest FU	MTC1 for homeless persons (*n* = 183) 12 month program MTC2: lower intensity, flexible program (*n* = 93) 12 month program Controls: treatment as usual (*n* = 66)	12 months	MTC2 had less alcohol intox* + fewer illegal drug use** + used less substances** than TAU No *≠* between MTC1 and TAU	No *≠* between MTC1 or MTC2 and TAU	MTC1*** and MTC2*** more likely to be employed than TAU	No *≠* between MTC1 or MTC2 and TAU regard. HIV risk behavior and psychological dysfunctions	
				24 months	MTC2 had less alcohol intox* + used less substances* compared with TAU No *≠* between TC1 and TAU	MTC1* and MTC2*** committed fewer crimes than TAU	MTC1** and MTC2*** more likely to be employed than TAU	MTC2 had less symptoms of depression*** and anxiety* than TAU	MTC2 improved more on several outcomes measures than MTC1 MTC completers scored sign better than MTC drop-outs and TAU

(11) French et al., 1999 (New York, USA) [50]	Prospective controlled study design (QES: sequential group assignment) Outcomes at last FU-point (on average 24 months after baseline)	342 homeless mentally ill substance abusers FU: 82%	MTC for homeless persons (*n* = 228) 12 month program Controls: treatment as usual (*n* = 53)		No *≠* regard. substance use outcomes	Fewer criminal activity**	Better employm. outcomes (ns)	Lower scores on BDI*, no *≠* regard. other psychological symptoms or risk behavior	

(12) Nuttbrock et al., 1998 (New York, USA) [38]	Prospective controlled study design (QES, as allocation based on availability + client preference) Outcomes 12 months after start Tx	290 homeless men with major mental disorder and history of substance abuse FU: not reported	Modified TC (*n* = 169) 18 month program Controls: 2 homeless community residences (*n* = 121) 18 month program	43% stayed 6 months in TC (versus 55%); 25% stayed 12 months (versus 37%)	4.1 versus 30.1% pos. urine tests*; SR alcohol use: 0 versus 14.3%*; SR marijuana use: 2.6 versus 2.9%; SR crack use: 7.7 versus 14.2*			Greater (ns) reductions in psycho-pathology (depression, anxiety, psychiatric distress) MTC participation predicted lower levels of anxiety** and better GAF-scores**	Drop-out after 6–12 months in community residences was predicted by substance use severity*

(13) McCusker et al. 1997 (New England, USA) [51]	Prospective controlled study design (no real randomisation, since *≠* interventions at both study sites) Outcomes 3 months after discharge and 18 months after admission	539 drug abusers entering residential Tx at 2 sites FU: 86% after 18 months	Traditional TC program (6 (*n* = 86) and 12 month alternative (*n* = 75)) Controls: MTC program (relapse prevention) 3 (*n* = 192) and 6 month (*n* = 186) alternatives	Tx completion: 23% in long TC, 34% in shorter TC, 31% in long MTC and 56% in short MTC	Time to drug use not *≠* between TCs and than MTC Stronger effect of long TC versus short TC and MTCs regard. drug and alcohol severity (ns)	Stronger effect of long TC versus short TC and MTCs regarding legal problems	Effect of TC on employm. stronger than in MTC*	Small effects of long TC versus short TC and MTCs regard. other ASI domains	

(13) McCusker et al., 1996 (Massachusetts, USA) [52]	Prospective randomized controlled study design Outcomes 6 months after Tx	444 drug abusers entering one residential Tx facility FU: 74%	Long MTC (*n* = 221) 6 month program Controls: short MTC (*n* = 223) 3 month program	Program completion: 30 versus 56% in short TC program	Relapse to drug use in first week after leaving Tx: 33 versus 70%* No group *≠* in heroin or cocaine use				Greater improvement in levels of depression among persons staying >80 days in TC*** Length of stay in TC** + program completion* pos. associated with levels of precontemplation Persons staying >80 days in TC had lower drug use***

(13) McCusker et al., 1995 (New England, USA) [53]	Prospective controlled study design Outcomes 3–6 months after discharge	628 drug abusers entering residential Tx at 2 sites FU: 84% in TC versus 74% in MTC	Traditional TC program (6 (*n* = 97) and 12 month (*n* = 87) alternative) Controls: MTC 3 (*n* = 223) and 6 month program (*n* = 221)	40 day retention: respectively, 70, 85, 73, and 72%; Tx completion: respectively, 33, 21, 56, 30% (ns *≠* in 4 groups)	Relapse: 50% in TC versus 44% in MTC No *≠* in number of days of drug use				

(14) Hartmann et al. 1997 (Missouri, USA) [43]	Controlled study design (QES, self-selection for exp. intervention) Outcomes at least 5 months after release	286 male offenders with a history of substance abuse No information on FU-rate	Prison TC graduates (*n* = 161) No information on program length Controls: comparison group of eligible persons who did not attend prison TC (*n* = 125)		No substance abuse: 67.4 versus 62% (ns)	No arrest: 85.4 versus 72%** Reincarc.: 16.4 versus 27.6*			

(15) Bale et al., 1984 (California, USA) [39]	Prospective controlled study design (only partial randomization due to substantial drop-out after group allocation) Outcomes after 2 years	363 male veterans addicted to heroin entering withdrawal Tx FU: 95.6%	3 TCs (*n* = 181): standard TC (*n* = 25) + two MTCs (*n* = 77 and *n* = 79) 6 month programs Controls: 5-day withdrawal Tx (*n* = 166)	Mean TIP longer in TC 1 (10.4 weeks) and TC3 (11.5 weeks)* than in TC2 (6.0 weeks)	No heroin use: 40, 48.1, and 35.4% versus 33.3% of controls; No other illegal drug use: 40, 41.6, and 53.3%* versus 39.3% of controls More alcohol problems: 40, 47.3** and 30.8% versus 22.4% of controls	No conviction: 44*, 32.5 and 59.5%** versus 31.3% of controls	Employed/attending school: 48*, 46.8 and 51.9%** versus 34% of controls	Mortality: 1.7% in TCs versus 6.6% among controls	The 3 TCs differed largely on program characteristics Heroin use + other major outcomes sign. better among subjects staying longer in Tx

(15) Bale et al., 1980 (California, USA) [40]	Prospective controlled study design (as treated analyses) Outcomes after 1 year	585 male veterans addicted to heroin entering withdrawal Tx FU: 93.2%	Veterans staying long (≥50 days) (*n* = 75) or short in TC program (<50 days) (*n* = 75) Controls: MMT (*n* = 59); detox only (*n* = 224); detox + other Tx (*n* = 112)	1-year retention rate: <5% in TC versus 74.5% in MMT	Recent heroin use + any illicit drug use lower in long TC subjects (37.3 and 29.3) than detox only-group (65.5 and 46.9)**, but not than MMT group (46.6 and 38.6%)	Arrest (37.3%), conviction (21.3%) and reincarc (4%) rate sign lower than in detox only-group (54.5**, 38** and 21.1%***, resp.), but not than MMT group (49.2, 22, and 10.2%)	Employm./school attendance: 65.3% of long TC group, 50.9% of MMT and 38.4%*** of detox only-group	Sign. more subjects had good global outcome score in long TC (64%) and MMT (54.45%) versus detox only-group (33.8%)	TC with confrontational style least successful Short TC group did not score sign better than detox only-group Twice the number of long TC subjects (29.3%) had the max. global outcome score than MMT clients (14%)

(29) Coombs, 1981 (California, USA) [41]	Prospective controlled study design (group allocation by self-selection) Outcomes 11–18 months after leaving TC	207 heroin addicts starting treatment in one of 2 TCs FU: 78.5%	Long-term TC (*n* = 77) 12 month program Controls: short-term TC (*n* = 130) 3 month program	Program completion: 63.6 versus 74.6%	Total abstinence: 4.3 versus 0%; Return to heroin use: 28.6 versus 53%				Program graduates used less often illicit drugs and were less likely to have relapsed or to be rearrested compared with splittees. Also higher employment rates among graduates

TC: therapeutic community, MTC: modified therapeutic community; SR: self-reported; QES: quasi-experimental study; Tx: treatment; TIP: time in program; BDI: Beck Depression Inventory; ASI: Addiction Severity Index; level of significance: *P < 0.05; **P < 0.01; ***P < 0.001.

**Table 2:** Summary of the findings from the selected studies (*n* = 16).

Reference number of the study/studies	Type of TC	Comparison condition	Followup length	Outcome measures					
				Retention	Substance use	Criminal activity	Employment	Health	Family and social relations
(1) Sacks et al., 2012 [19]	Prison	TAU	1 year			+			

(2) Zhang et al., 2011 [44]	Prison	TAU	1 year			=			
			5 years			=			

(3) Messina et al., 2010 [45]	Prison	Other TC	1 year	+	=	=		=	=

(4) Welsh, 2007 [37]	Prison	TAU	2 years		=	+	+		

(5) Sullivan et al., 2007 [46]	Prison	TAU	1 year		+	+			

(6) Morral et al., 2004 [47]	Prison	TAU	1 year	=	+	=		+	

(7) Inciardi et al., 2004 [28]	Prison	TAU	6 months		+	+		+	
			1 year		+	+			
			3 years		+	=			
			3 years 6 months		+	+			
			5 years		+	+			

(8) Prendergast et al., 2004 [33]	Prison	TAU	1 year		+	+			
			2 years			+			
			5 years	=	=	+	=	=	

(9) Greenwood et al., 2001 [25]	Community-based	Other TC	6 months	=	+			+	+
			1 year	=	=			+	
			1 year 6 months		=			+	+

(10) Nemes et al., 1999 [42]	Community-based	Other TC	1 year 6 months	=	+	+	+		

(11) De Leon et al., 2000 [24]	Community-based	TAU	1 year		+	=	+	=	
			2 years		+ =	+	+ =	+	

(12) Nuttbrock et al., 1998 [38]	Community-based	TAU	1 year	−	+			+	

(13) McCusker et al., 1997 [51]	Community-based	Other TC	6 months	=	=				
			1 year	−	=	=	+		

(14) Hartmann et al., 1997 [43]	Prison	TAU	6 months		=	+			

(15) Bale et al., 1984 [39]	Community-based	TAU	1 year	−	+	+	+	+	
			2 years	+	+ (illicit) − (alcohol)	+	+		

(16) Coombs et al., 1981 [41]	Community-based	Other TC	1 year	=	+				

TC: Therapeutic Community, Other TC: Other TC modality, TAU: Treatment As Usual.
